# The grand escape – how pathogens outsmart the human complement system^☆^

**DOI:** 10.1016/j.imbio.2025.153126

**Published:** 2025-10-24

**Authors:** A.A. Nowacka, L. Sordo Vieira, V. Petr, B. Fageräng, R. Würzner, M. Ohms

**Affiliations:** a Institute for Systemic Inflammation Research, University of Lübeck, Lübeck, Germany; b Division of Pulmonary, Critical Care, and Sleep Medicine, Department of Medicine, University of Florida, Gainesville, FL, United States of America; c Department of Nephrology, Institute for Clinical and Experimental Medicine, Prague, Czech Republic; d Department of Immunology, University of Oslo, and Oslo University Hospital, Oslo, Norway; e Department of Clinical Immunology, Rigshospitalet, and Faculty of Health and Medical Sciences, University of Copenhagen, Copenhagen, Denmark; f Institute of Hygiene & Medical Microbiology, Medical University of Innsbruck, Innsbruck, Austria

**Keywords:** Immune evasion, Complement, Pathogen, Infection, Biomarkers, Therapy

## Abstract

Infectious diseases remain a significant cause of mortality and morbidity worldwide. Complement is a critical component in the defense against pathogens and despite their great differences, viruses, bacteria, fungi, and protists have all developed similar mechanisms of evasion from the human complement system. Using examples from four microbial groups (viruses, bacteria, fungi and protists), this review expands on examples of these different mechanisms of evasion. The mechanisms are grouped as (A) avoidance of recognition, (B) avoidance of eradication, (C) avoidance of activation and function, or (D) use of the complement proteins for entry into the host, in accordance with the classification initially proposed in 1999. Furthermore, this review will expand on novel descriptions of complement evasion, for example involving intracellular complement. Taken toge complement evasion is an essential tool used by pathogens not only in a defensive manner, protecting the pathogen from the host, but can also employed in an aggressive manner to aid the invasion of the host. Understanding these mechanisms has already influenced diagnostic and therapeutic tools, including vaccine development, and a further expansion of evasion molecules as biomarkers, vaccines or targets for therapy appears likely in the future.

## 1. Introduction

The complement system is essential for many biological functions, among the most important is host defense against pathogens (Merle et al., 2015a; Merle et al., 2015b). Not only does complement have the ability to kill bacteria directly by damaging their cell membranes (Xie et al., 2020), but it also enhances phagocytosis by opsonization, increases immune cell recruitment by chemotaxis, and regulates the greater immune state of the host (Morgan, 1999). The importance of the complement system can be demonstrated by the large number of evasion strategies that pathogens have developed during their co-evolution with humans.

The first comprehensive review reviewing complement evasion mechanisms across all four major kingdoms – viruses, bacteria, fungi and protists – was published in 1999 (Würzner, 1999). Before then, only single groups of microorganisms or even only single pathogens had been looked at. This review from 1999 introduced the concept of dual categorisations. This categorisation classed these mechanisms into two parts, where most pathogens aim to primarily avoid recognition (reviewed in section A), but upon recognition can employ an arsenal to avoid destruction (reviewed in section B-D).

However, since 1999 new subclasses and examples of mechanisms have been proposed, especially for the latter, where the invader can ignore any protective measures, and instead employs its armamentarium to invade the host (reviewed in section D2-D3). Furthermore, a new subcategory has only very recently become evident. This category introduces intracellular complement as a target of evasion (reviewed in section B6).

This review will focus on different mechanisms though which pathogens can evade complement. We have employed two approaches for our review. One was to study the most recent reviews on this topic and the cited original references therein. The second approach involved conducting a broad literature search in PubMed and Google Scholar using the terms “complement AND evasion”, along with related keywords to ensure comprehensive coverage. We systematically examined both review articles and original research papers, not only to gather established findings but also to identify recurring themes, overlooked mechanisms, or emerging concepts. When a particular aspect offered a novel angle, filled a knowledge gap, or challenged existing understanding, we pursued it in greater depth to gain a more nuanced perspective on the topic.

## 2. Complement in immune defense against the invader

### 2.1. Activation of complement

Description of complement activation and regulation is out of scope of this review, and we refer the reader to comprehensive papers published elsewhere (Merle et al., 2015a). In brief, the complement system can be activated by three pathways, the classical (CP), lectin (LP) and alternative (AP) pathway. Each pathway has a distinct initiating mechanism that allows recognition of a broad spectrum of structures.

The CP is initiated by the binding of of the C1q molecule to pre-bound immunoglobulins (Ig), IgM, and IgG, or specific pathogen-associated molecular patterns (PAMPs). LP activation occurs through the interaction of mannose-binding lectin (MBL), ficolin-1, -2 or -3, or collectin-11 with carbohydrate moieties displayed on the pathogen surface. The activation of the CP and LP leads to the cleavage of C2 into C2a and C2b, and C4 into C4a and C4b, and the subsequent formation of the C3 convertase C4b2a. The AP is activated by the spontaneous hydrolysis of a labile thioester bond present in the C3 molecule, generating a biologically active conformation, C3(H_2_O). C3(H_2_O) then associates with factor B (FB) and following an interaction with factor D (FD), to form the C3 convertase C3(H_2_O)Bb.

All three complement pathways converge at the formation of a C3 convertase, which exists in two main forms: the C3bBb and the C4b2a. The convertases cleaves C3 into C3a and C3b, which binds covalently to surfaces with exposed hydroxyl groups, such as microbial surfaces. Binding of C3b to a surface stabilizes the molecule and increases complement activation through the amplification loop.

C3b then joins the enzymatic complexes, forming the C5 convertases (C3bBbC3b or C4b2aC3b). The C5 convertase cleaves the inactive C5 molecule into two active fragments: the anaphylatoxin C5a and the bioactive fragment C5b, and thereby initiates the terminal pathway. C5b sequentially binds to C6, C7, and C8, followed by several C9 molecules. This assembles the membrane attack complex (MAC), also known as C5b-9 or the terminal complement complex (TCC). The C5b-8 complex is embedded into the target membrane, after which C9 molecules polymerize to form a pore, leading to calcium influx and lysis of the pathogen (Merle et al., 2015a). Further details on MAC regulation are discussed below.

## 3. Complement effector functions

Complement is highly capable at preventing infection by invading pathogens and is a central component to both the adaptive and innate immune responses. Complement effector functions include, but are not limited to:

- Opsonization of microbes to improve phagocytosis
- Chemoattraction of immune cells to sites of infection
- Formation of membrane attack complex (MAC) on microbe membranes
- Activation of phagocytes to improve immune functions
- Connection of the innate and adaptive immune system

### 3.1. Opsonization

Complement-mediated opsonization is a key immune process in which activated fragments of complement proteins – primarily C3b, iC3b, and C4b – bind covalently to microbial surfaces, typically glycoproteins. This deposition effectively “tags” microbes for recognition by the immune system. These complement fragments act as opsonins, facilitating the engagement of complement receptors (CR1, CR3, and CR4) on phagocytic cells such as macrophages and neutrophils. This interaction promotes phagocytosis and results in the clearance of the microbe (Vandendriessche et al., 2021; Lambris et al., 2008).

### 3.2. Chemoattraction

Chemoattraction is the movement of cells in the direction of increasing concentrations of a signaling molecule. This occurs in immunity, in which immune cells are attracted to an area of inflammation through the binding of various signaling molecules, including complement fragments (Fernandez et al., 1978). Complement activation generates the anaphylatoxins, C3a and C5a, which are very potent chemoattractants. Anaphylatoxins bind to the receptors C3aR, C5aR1 and C5aR2 and can induce the migration of leukocytes expressing the C3aR and C5aR1 receptors (Klos et al., 2009). Both C3a and C5a are chemotactic for granulocytes (neutrophils, eosinophils and basophils), monocytes/macrophages and mast cells. In addition, C5a is also chemotactic for dendritic cells (DCs), germinal center B cells and T cells (Klos et al., 2009).

### 3.3. Mechanisms of MAC

The MAC is a crucial component of the immune system’s defense against pathogens, particularly bacteria. MAC formation begins when the complement system is activated on the surfaces of pathogens, resulting in the cleavage of complement component C3 and eventually C5. The C5b forms a stable C5b-6 complex upon binding to C6, which then associates with C7, the limiting molecule for this assembly (Würzner, 2001), to create a firmly attached C5b7 complex on the membrane (Merle et al., 2015a). The subsequent recruitment of C8 causes membrane protrusions and forms a binding site for C9 (Brannen and Sodetz, 2007). Once the first C9 molecule integrates into the C5b–8 complex, additional C9 molecules join, forming a pore that disrupts the bacterial cell membrane and induces lysis (Ramm et al., 1982).

The MAC is traditionally known for its ability to lyse gram-negative bacteria. Gram-negative bacteria are particularly vulnerable to MAC lysis due to the outer membrane and their relatively thin peptidoglycan layer. Gram-positive bacteria, on the other hand, have long been considered resistant to MAC-mediated lysis because of their thick peptidoglycan walls. However, this view is becoming increasingly challenged. Recent studies have shown that MAC components can assemble on Gram-positive bacteria at certain sites, and that the effectiveness of this assembly varies among bacterial strains (Berends et al., 2013). In fact, deficiencies in MAC components have been shown to be associated with increased susceptibility to infections, not only by Gram-negative bacteria but also by certain Gram-positive bacteria (Skattum et al., 2011).

Some Gram-positive species, such as *Streptococcus pyogenes*, have evolved mechanisms to evade MAC formation – for example, by producing proteins that block essential steps in the pore formation (Akesson et al., 1996). This demonstrates that the thick peptidoglycan barrier alone is not necessarily sufficient for protection against MAC-induced damage (Akesson et al., 1996). Furthermore, the classical notion that the MAC simply forms pores stretching through bacterial envelopes to cause cell death is still debated; evidence suggests that the mechanism may not involve complete membrane penetration in all cases (Mayer, 1972). Local assembly of the MAC at the bacterial surface is vital for effective bactericidal activity, with membrane-bound C5 convertases playing a critical role in generating MAC pores that can penetrate the bacterial membranes (Doorduijn et al., 2019). Membrane-bound C5 convertases are crucial for forming bactericidal MAC pores because they cleave C5 directly on the bacterial surface, enabling the unstable C5b fragment to rapidly bind to C6 and C7. This immediate, localized assembly maintains C5b’s active state and supports effective MAC insertion. Preassembled C5b6 formed in solution lacks this coordination and quickly becomes non-functional (Heesterbeek et al., 2019).

Recent findings emphasize that for effective bacterial killing, MAC must damage both the outer membrane and inner membrane of gram-negative bacteria, highlighting the intricate nature of bacterial defenses against immune attacks (Doorduijn et al., 2019). Overall, the assembly and function of MAC pores are essential for robust immune responses against a wide range of bacteria.

### 3.4. Activation of phagocytes

The anaphylatoxins bind to a broad spectrum of immune and non-immune cells. They activate mast cells and basophils to release histamine and they trigger an oxidative burst in macrophages, neutrophils, and eosinophils. Moreover, they regulate vasodilation, increase the permeability of small blood vessels, and induce smooth muscle contractions (Yasuda et al., 2023). Activated neutrophils play a crucial role in the innate immune response, particularly through their interaction with complement factors. Among the complement cleavage products, C5a and C5a des-Arg significantly enhance neutrophil functions, while C3a and C4a show minimal effects. These active fragments stimulate changes in membrane potential, intracellular pH, glucose uptake, and cellular size, leading to increased phagocytosis and reactive oxygen species (ROS) generation (Vandendriessche et al., 2021; Morris et al., 2011; Messerer et al., 2018). C5a also modulates the expression of critical neutrophil activation markers and receptors, including C5aR1, CD62L, CD10, and CD11b (Wohlgemuth et al., 2022). The C5a response is characterized by rapid kinetics, with distinct phases observed for depolarization and alkalization, as well as delayed upregulation of markers like CD11b and CD16 (Wohlgemuth et al., 2022). Concentration-dependent studies of C5a levels indicates its binding to specific receptors, with effective concentrations in the nanomolar range, aligning with inflammation-related levels (Wohlgemuth et al., 2022). Importantly, the activation of neutrophils by C5a does not promote platelet-neutrophil complex formation, suggesting a different mechanism of complement-mediated activation (Wohlgemuth et al., 2022).

### 3.5. Linkage to the adaptive immune system

The complement system has important roles in T cell biology, including the regulation of metabolism, regulation of activation thresholds, receptor expression, and differentiation. For example, simultaneous signaling through pattern recognition receptors and an anaphylatoxin receptor on antigen presenting cells, favors a type 1 T helper response (Th1). The intracellular complement system (complosome) affects cell physiology through direct cross-talk with other intracellular innate sensor systems – so called inflammasomes (Triantafilou et al., 2016; Arbore and Kemper, 2016). This regulates, for example, human Th1 responses by driving the signaling pathways and metabolic reprogramming necessary to induce effector responses (Pavlov et al., 2008; Strainic et al., 2008; Lalli et al., 2008; Heeger et al., 2005; Song, 2012; Liu et al., 2005; Kolev et al., 2015; Hess and Kemper, 2016). Furthermore, complement controls the induction and contraction of T cell responses (Dunkelberger and Song, 2010; Clarke and Tenner, 2014; Kolev et al., 2013). Complement’s regulatory effects can therefore be mediated directly (by modulating the T cell itself) and/or indirectly (by affecting the function of antigen presenting cells) (West et al., 2018).

Additionally, a recent study found that the T cell-dependent B cell maturation process is dependent on C3a and C5a signaling. Specifically, germinal center B cells downregulate their surface inhibitors except for CD59, which increases complement activation on their surface while still protecting them from cell lysis (Cumpelik et al., 2021). T cell-dependent antibody production was reduced in C1q-deficient mice, suggesting the importance of the CP. T cell-independent B cell responses may also be dependent on anaphylatoxin signaling and in particular AP activation (Cumpelik et al., 2023). Of note, an interaction between C3 fragments and complement receptors influences the threshold for B cell activation (Melchers et al., 1985; Kulik et al., 2011; Kovács et al., 2021).

## 4. Complement regulators

The complement system’s powerful functions can harm the host, especially when activated inappropriately during conditions like tissue ischemia and reperfusion. To prevent damage to host cells while effectively targeting foreign materials, mammals have developed various inhibitory proteins to regulate complement activity.

C1 inhibitor (C1INH) inactivates C1r and C1s in the CP and MASP-1 and MASP-2 in the LP, thus inhibiting the initiation of these two pathways (Davis et al., 2010). Complement factor H (FH) and complement factor I (FI) are key regulators of the AP, with FI inactivating C3b by forming iC3b in the presence of cofactors such as FH (Roversi et al., 2011). FH also competes with FB for binding to C3b and therefore accelerates the decay of the C3 convertase complex (Ferreira et al., 2010). Membrane cofactor protein (MCP, CD46) acts as a cofactor for FI, facilitating the cleavage of C3b and C4b (Liszewski et al., 1991). Decay-accelerating factor (DAF, CD55) enhances the dissociation of C3 convertases of all three pathways (Hourcade et al., 2002). CR1 acts by removing opsonized immune complexes and has a cofactor activity for FI (Khera and Das, 2009). Thrombomodulin enhances FI-mediated inactivation of C3b and can des-arginate anaphylatoxins, further modulating complement activity (Delvaeye et al., 2009). Vitronectin binds to C5b-7 and C9, preventing their incorporation into membranes and inhibiting polymerization (Preissner et al., 1989). Clusterin binds to C5b-7, C8 and C9 and similarly inhibits polymerization without completely preventing MAC assembly (Tschopp et al., 1993). CD59 is a key regulator of the terminal pathway and blocks the association of C9 with C5b-8 to prevent MAC formation (Farkas et al., 2002).

## 5. Complosome

In recent years, novel insights into complement biology have unveiled the concept of cell-autonomous and intracellular complement, termed the “complosome” (Arbore et al., 2016). This emerging paradigm positions the complosome as a crucial orchestrator of normal cell physiology, influencing essential processes such as mitochondrial activity, glycolysis, oxidative phosphorylation, cell survival, and gene regulation. Its impacts extend beyond immune cells, encompassing non-immune cells like fibroblasts, as well as endothelial cells (Mahajan et al., 2021) and epithelial cells (Kulkarni et al., 2019). Given these roles, the complosome emerges as a central player in maintaining cell homeostasis and regulating effector responses. The comprehensive review by West and Kemper (West and Kemper, 2023) serves as an excellent resource, providing detailed information on the significance of intracellular complement in cellular functions.

Additionally, C3 functions as a sensor for intracellular pathogens, activating cell-intrinsic immunity when a C3-bound pathogen enters the cells (Brock and Parmely, 2017). For instance, C3 bound to *Listeria monocytogenes* during an infection of intestinal epithelial cells, facilitates the clearance of pathogens by inducing autophagy of the host intestinal epithelial cell (Sorbara et al., 2018). These pieces of evidence demonstrate intracellular roles for C3 when activated C3/C3b is brought into the cytosol by invasive pathogens. Evasion mechanisms related to intracellular complement have not yet been extensively described.

### 5.1. Avoidance of recognition – disguise

#### 5.1.1. Disruption of antibody-complement interactions

**Fig. 1.**
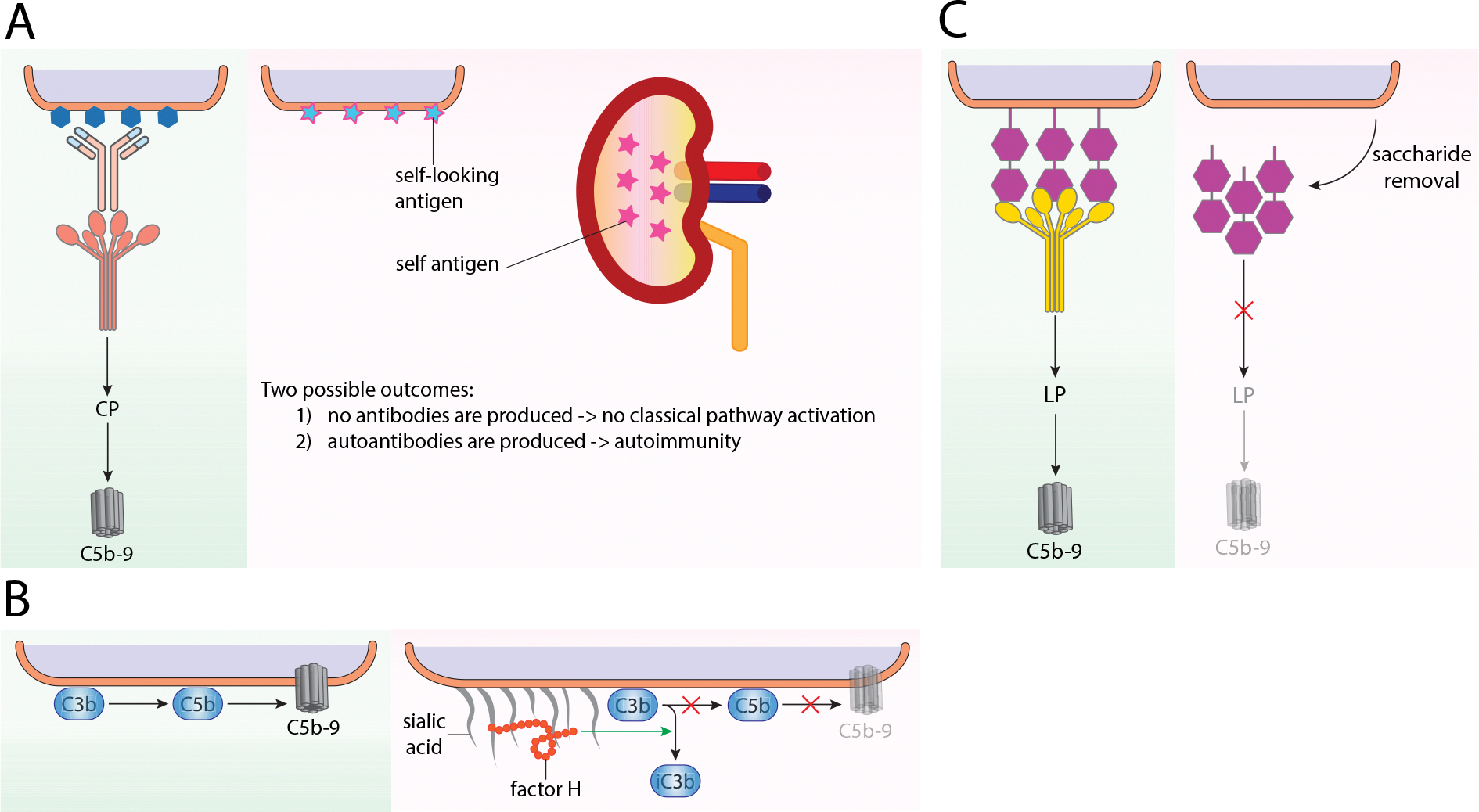
Avoidance of recognition–disguise. (A) Under normal conditions, activation of the CP via antibody-antigen interactions with C1q lead to proper clearance of pathogens. However, some pathogens, such as Streptococcus pyogenes, can present antigens that resemble host antigens, thus disguising themselves as self; a key example is the M protein, a major virulence factor that mimics host antigens through molecular mimicry, particularly in its N-terminal region, enabling the bacteria to evade the host’s immune system by appearing similar to host tissues. (B) Complement activation leads to the potent activation of the membrane attack complex. Sialic acid enhances the binding of FH to host cells (and thus complement inhibition), and thus the decoration of host’s sialic acids either by stealing or de novo synthesis is a common strategy of complement regulation. (C) Modulation of complement-activation molecules from pathogen’s surface is another common mechanism to evade complement attack. For example, *K. pneumonia* lacking mannose-poor O antigens are less susceptible to killing by polymorphonuclear leukocytes.

Through evolution, pathogens have developed strategies to avoid recognition by the complement system. The ideal solution for pathogens is to avoid immune detection in the first place. Examples of structural barriers that help to disguise pathogens can be found in Table 1. A commonly used feature is molecular mimicry, defined as the sharing of antigenic determinants between the pathogen and host (Fishelson, 1991). The pathogen is thus recognized by the host’s immune system as a self-structure and limited immune response is triggered. Molecular mimicry strategies are also well described causes of autoimmunity (Cusick et al., 2012). For example, the M protein of *S. pyogenes* elicits the formation of autoantibodies that cross-react with heart myosin which can cause heart damage (Fig. 1A) (Whitton and Feuer, 2004).

Antibodies are responsible for many essential functions in the immune system, and as such many pathogens attempt to avoid antibody recognition. There is therefore a strong selection pressure for pathogens to conceal neutralizing epitopes from human antibodies. Common mechanisms of antibody escape include antigenic drift, epitope shielding and immune redirection to dominant but non-protective epitopes (Qerqez et al., 2023). One strategy to evade antibody binding is the expression of highly immunogenic but non-protective decoy epitopes (Hsieh et al., 2021). The primary characteristics of decoy epitopes involve redirecting the humoral response away from important epitopes and promoting the production of non-neutralizing antibodies against non-essential epitopes. Another way to protect neutralizing epitopes is by glycosylation. For instance, the human immunodeficiency virus 1 (HIV-1) envelope is decorated with >25 *N*-linked glycosylation sites that shields neutralizing epitopes (Kim et al., 2015). Another strategy is the structural blockade of neutralizing epitopes. In severe acute respiratory syndrome coronavirus (SARS-CoV) -1 and -2 the cross-reactive antibody, CR3022, binds a cryptic epitope that is revealed only in a specific conformation of receptor-binding domain (RBD). This way the epitope is concealed from CR3022 until binding to the ACE2 receptor (Yuan et al., 2020).

Another mechanism to prevent antibody binding is the expression of Fc receptors or Fc-binding proteins on the pathogen surface. These molecules engage the Fc region of human IgG, which thus blocks antibody-dependent mechanisms. For instance, viruses belonging to the families Herpesviridae and Coronaviridae express receptors that result in the binding of non-specific IgG to the viruses or virus-infected cells, resulting in the steric hindrance of virus-specific immune IgG (Dowler and Veltri, 1984). In some cases an antibody may bind to both the surface epitopes and viral Fc receptors, called antibody bipolar bridging effect, which hinders virus binding. This effectively inhibits most antibody-dependent immune mechanisms (Frank and Friedman, 1989). Some pathogens can also clear antibody-antigen complexes from their surfaces as in the case of alphaherpesvirus pseudorabies virus (PRV) (Favoreel et al., 1999), or parasites such as *Trypanosoma* (Balber et al., 1979; Frevert and Reinwald, 1990).

#### 5.1.2. Hiding of surface molecules crucial for complement recognition

Several pathogens evade complement attack by masking the moieties on their surface that lead to complement activation in the host. Hiding from antibodies is described in section A1.

For example, the binding of FH or the prevention of IgM binding to host cell surfaces due to presence of glycosaminoglycans may inhibit complement activity (Dudek et al., 2022). Nontypeable *Haemophilus influenzae* strains show that a defect in their ability to sialylate LPS leads to less virulent strains. This effect is shown to be FH independent (Figueira et al., 2007) in contrast to the FH mediated effect of sialylated *Neisseria gonorrhoeae* (Ram et al., 1998). As such, several bacteria have adapted strategies to either synthesize their own sialic acids or utilize host’s machinery to sialylate their surfaces, pretending to be host by molecular mimicry (Fig. 1B) (Vimr et al., 2004).

Mutants of the fungal *Aspergillus* species lacking genes associated with pigmentation, display higher complement deposition on its surface, suggesting a role for melanin in the masking of complement-activating moieties (Speth and Rambach, 2012; Tsai et al., 1997; Tsai et al., 1998).

#### 5.1.3. Capsules as barrier against complement

Capsules shield bacteria from recognition and destruction by the host immune response. The capsule of *Neisseria meningitidis* prevents antibody mediated C1q recruitment to the bacterial surface and inhibits the initiation of the CP (Agarwal et al., 2014). The capsule also masks subcapsular targets for C3b deposition, resulting in the interruption of the AP amplification loop (Roberts, 1996). The complex structure of the capsule therefore sterically hinders the binding of C3b molecules to complement receptors on immune cells.

Furthermore, some bacteria like *Klebsiella pneumonieae*, modify their capsule composition by removing rhamnobiose and mannobiose, thereby inhibiting recognition by the LP (Fig. 1C) (Sahly et al., 2009). The lipooligosaccharide (LOS) in *N. gonorrhea* contains a lacto-N-neotetraose moiety which can be sialylated by the enzyme LOS sialyltransferase (Gilbert et al., 1996). The sialylation of LOS interferes with IgG binding, MBL recognition, and represses AP activation through enhanced FH binding, and therefore inhibits all three complement pathways (Elkins et al., 1992; Gulati et al., 2002).

Effective complement activation is crucially dependent on proximity to the pathogen. For instance, IgG-C1q assembly relies on optimal antigen epitope distribution, and proximity is necessary for subsequent CP activation (Diebolder et al., 2014). In addition to inhibiting the binding of C1q and other complement components to the bacterial surface, the elongated O-antigen of *Klebsiella pneumoniae* inhibits the terminal complement pathway since any deposited C3b is too far away from the membrane to allow further activation or stabilization at the surface (Merino et al., 1992).

*Acinetobacter baumannii* is a Gram-negative bacterium renowned for its exceptional resistance to complement-mediated killing, a key aspect of its serum resistance. A central protective structure is its exopolysaccharide capsule, which is composed of long-chain polysaccharides formed from repeating carbohydrate units, and its synthesis is governed by genes located in the capsule locus. To date, over 128 capsule locus types have been identified, contributing to significant structural and compositional diversity of capsular polysaccharides among *A. baumannii* strains (Magda et al., 2025). This structural variability plays a critical role in immune evasion. The capsule acts as a physical shield, preventing the effective deposition or insertion of complement components like C3b, C4b, and the MAC into the bacterial surface. Notably, although MAC deposition can occur, especially in some capsule types, *A. baumannii* often survives despite MAC presence, suggesting incomplete insertion or interference with pore formation (Monem et al., 2020).

### 5.2. Avoidance of eradication – removal

#### 5.2.1. Removing complement fragments from the pathogen’s surface

**Fig. 2.**
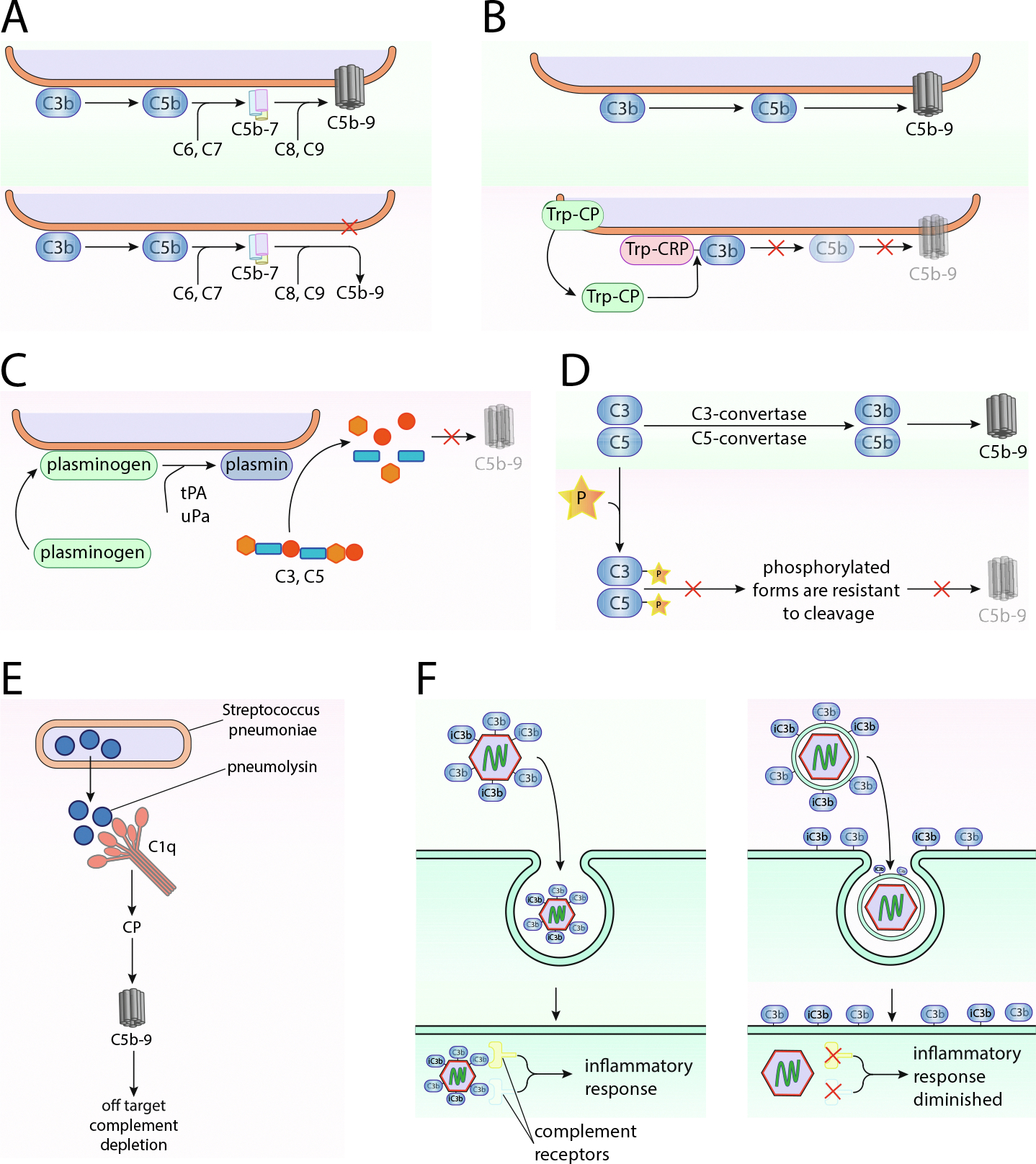
Avoidance of eradication–removal. (A) Serum-resistant *Salmonella minnesota* S218 sheds the MAC from its surface after addition of C8 and C9 as the nascent complex cannot insert into the hydrophobic outer membrane of the pathogen. (B) *Trypanosoma cruzi* displays CRP which binds C3b there and the CRP-C3b complex is then cleaved by the parasitic cysteine protease CP downmodulating complement activation. (C) Pathogens can hijack host proteases to attenuate complement activation, e.g. plasminogen via parasitic plasminogen binding proteins. Once bound, plasminogen can be activated by either human or microbial activators to its active form, plasmin, which cleaves C3 and C5 via the action of C5 and C3 convertases. (D) Leishmanial surface protein kinase LPK-1 can phosphorylate C3 and C5, thereby inhibiting their cleavage. (E) *S. pneumoniae* pneumolysin is released and then causes complement activation away from the surface of the pathogen. (F) Complement coated non-enveloped viruses induce intracellular complement sensing leading to inflammation, whereas enveloped viruses, such as RSV, leave the C3b on the outside of the cell when engulfed, resulting in a diminished complement activation.

In case the pathogen is unable to avoid recognition, complement fragments successfully deposit on its surface (Table 2). Many pathogens, however, have acquired the ability to shed these fragments to avoid destruction. A serum-resistant strain of *Salmonella* (*Salmonella minnesota* S218) removes the MAC from its surface after addition of C8 and C9 (Fig. 2A) thanks to an inability of the complex to insert into the hydrophobic outer membrane of the pathogen (Joiner et al., 1982).

#### 5.2.2. Protease-dependent mechanisms of complement evasion

The neutralization of complement components through degradation into smaller, non-functional fragments represents a key evasion strategy often utilized by bacteria. Two mechanisms for proteolytic cleavage of complement proteins are described. Firstly, the disruption of the complement cascade through pathogen-expressed proteases. Secondly, the acquisition and activation of host-derived plasminogen for indirect plasmin-mediated complement degradation (Ermert et al., 2019).

Pathogenic proteases have a wide range of substrates in the complement cascade. The cleavage of immunoglobulin and C1q by e.g., *Pseudomonas* elastase (PaE) or alkaline protease (PaAP), interferes with the activation of the CP by preventing the formation of ordered IgG Fc hexamers. This, in turn, compromises the structural platform needed for strong Fc–C1q interactions, effectively blocking downstream complement activation (Rooijakkers and Van Strijp, 2007).

Many pathogens degrade C3 as a central component of the complement cascade, circumventing the downstream complement functions like opsonization, phagocytosis, signaling through anaphylatoxin receptors and MAC formation. *S. pyogenes* exotoxin B (SpeB), a cysteine protease and an important virulence factor in Group A Streptococcus (GAS) infections, degrades serum C3 and enables GAS to resist complement damage, opsonization and prevents phagocytosis (Kuo et al., 2008). The cleavage of C3 by the autotransporter NaIP from *Neisseria meningitidis* occurs four amino acids upstream from the natural C3 cleavage site and produces shorter C3a-like and longer C3b-like fragments (Del Tordello et al., 2014). The C3b-like fragment has higher affinity for degradation by host complement regulators, FH and FI, and does not lead to C3b deposition on the bacterial surface (Del Tordello et al., 2014).

*Staphylococcus aureus* relies on a whole arsenal of virulence factors to escape the host immune response including the six serine-protease-like proteins, SplA to SplF. Notably, SplB functions as a potent and broad-spectrum protease that targets multiple complement components, effectively compromising the integrity of the entire complement system (Dasari et al., 2022). It inactivates C3, C4 and activation fragments C3b and C4b by preferentially cleaving their alpha chains. Moreover, it can cleave the components of the terminal complement pathway, C5, C6, C7, C8 and C9 (Dasari et al., 2022). Thus, through the expression of one multifunctional protease *S. aureus* escapes two major functions of the complement system, the C3b-mediated phagocytosis as well as the MAC deposition on the bacterial surface.

Not only bacteria evade complement activation through protease expression. The presence of the complement-degrading fungal serine protease Alp1 was shown in the supernatant of *Aspergillus fumigatus* cultures in cerebrospinal fluid (CSF) (Rambach et al., 2010). Alp1 has a cleavage affinity for the complement proteins C1q, C3, C4, C5, MBL, and FD (Rambach et al., 2010; Behnsen et al., 2010).

*Trypanosoma cruzi* displays a surface complement regulatory protein (CRP) which binds C3b. It is believed that the CRP-C3b complex is then cleaved by the parasitic cysteine protease reducing both C3 convertase formation and C3b opsonization on the pathogen’s surface (Fig. 2B) (Norris, 1996a; Norris, 1996b).

#### 5.2.3. Use of enzymes of the coagulation cascade for evasion

In addition to host-derived complement regulators, pathogens can also hijack host proteases to attenuate complement activation, e.g. plasminogen via plasminogen binding proteins. Once bound to such a microbial receptor, plasminogen becomes accessible and can be activated by either human or microbial activators resulting into its active form, plasmin. Plasmin suppresses complement at the level of C3 and C5, by cleaving these central proteins (Fig. 2C) (Potempa and Potempa, 2012). Plasminogen is, as a component of the coagulation system, abundant and thus omnipresent in the circulation, turning it into an easily accessible target for many human pathogens including: *Haemophilus influenzae* (Barthel et al., 2012), *Pseudomonas aeruginosa* (Kunert et al., 2007), *Borrelia burgdorferi* (Brissette et al., 2009), *Streptococcus pneumoniae* (Bergmann et al., 2004), *S. aureus* (Koch et al., 2012), and *Candida albicans* (Luo et al., 2009). Another well studied example for plasminogen acquisition and activation is *Leptospira*. At least eight proteins act as plasminogen-binding receptors of *Leptospira*, including the major outer membrane protein, LipL32 (Vieira et al., 2010). Activation of plasminogen either happens through microbial proteins on the surface of *Leptospira* or host-derived activators, like urokinase-type plasminogen activator (uPA) (Vieira et al., 2009). The bacteria-associated plasmin prevents IgG and C3b deposition on the leptospiral surface through cleavage and increases survival upon infection.

The malaria parasite, *Plasmodium falciparum*, also binds human plasminogen which then gets converted to plasmin, and leads to the cleavage of C3b, ultimately inhibiting MAC activity (Reiss et al., 2021).

#### 5.2.4. Chemical modifications of complement proteins

In addition to protease or peptidase cleavage, some pathogens have also acquired the ability to inhibit complement by chemical modifications. For example, the *Leishmanial* enzyme LPK-1, an outward facing surface protein kinase, which is rare in eukaryotic cells, can cause C3 and C5 phosphorylation, which is believed to inhibit cleavage of these proteins (Fig. 2D) (Hermoso et al., 1991).

#### 5.2.5. Distracting complement activation off the microbe

A secreted and then extracellular cysteine protease (SCP) of *S. pyogenes* leads to the activation of complement in the surroundings of *S. pyogenes,* but decreased complement deposition on the surface of the bacteria (Berge et al., 1997). As a secondary mechanism of *S. pyogenes*, SCP also releases a C5a peptidase that diminishes the effects of complement activation in the vicinity of the bacteria (Berge and Björck, 1995). This is similar to the effects of pneumolysin from *S. pneumoniae* which also causes complement activation away from the surface of the microbe (Fig. 2E) (Mitchell and Andrew, 1997).

In one study, galactosaminogalactan (GAG), a molecule secreted by *Aspergillus* during co-culture with human platelets, was shown to induce complement activation and deposition on the platelets, thereby reducing their viability. However, it remains unclear whether this process also led to reduced complement deposition on the *Aspergillus* itself, as this aspect was not addressed in the study (Deshmukh et al., 2020). Platelet loss can potentially lead to a higher hemorrhage and consequently to more access to free heme for the fungus, a key source of iron.

*Trypanosoma brucei* evades host immunity through the rapid internalization and recycling of its dense coat of variant surface glycoproteins (VSG). This high turnover is crucial for the removal of host immune effectors, including antibodies and other covalently bound complement proteins. These proteins are targeted for degradation and thus prevented from recycling back to the surface. In doing so, the parasite minimizes effective opsonization and complement-mediated lysis, thereby avoiding recognition and clearance by macrophages during chronic infection. In addition, the constant shedding of VSG molecules acts as a decoy mechanism, diverting complement activation away from the parasite and leading to ineffective complement consumption and potential hypocomplementemia (Onyilagha and Uzonna, 2019).

#### 5.2.6. Evading attack of intracellular complement

The mechanism of intracellular complement sensing is now recognized as a potent anti-microbial mechanism in mammalian cells to sense pathogens that have been tagged by complement and internalized (Tam et al., 2014). Enveloped viruses, such as respiratory syncytial virus (RSV), are believed to be resistant to intracellular complement sensing since their opsonization in the extracellular space should occur on the lipid membrane and thus be left behind in the process of membrane fusion (Fig. 2F) (Tam et al., 2014). Another important mechanism to evade intracellular complement sensing is by the expression of C3 degrading proteases by viruses, such as human rhinovirus or poliovirus 2 (Tam et al., 2014). This then downregulates opsonization or neutralization in the extracellular space and may also prevent intracellular sensing once internalized. This appears to be a common evasion strategy among viruses.

### 5.3. Avoidance of activation and function – Inhibition

#### 5.3.1. Interruption of complement activation

The most direct strategy to abrogate complement activation lies in the interruption of the specific protein-protein interactions that orchestrate the complement cascade. For this purpose, many pathogens express proteins that act as complement inhibitors that can either block, catch or destabilize complement (Table 3).

**Fig. 3.**
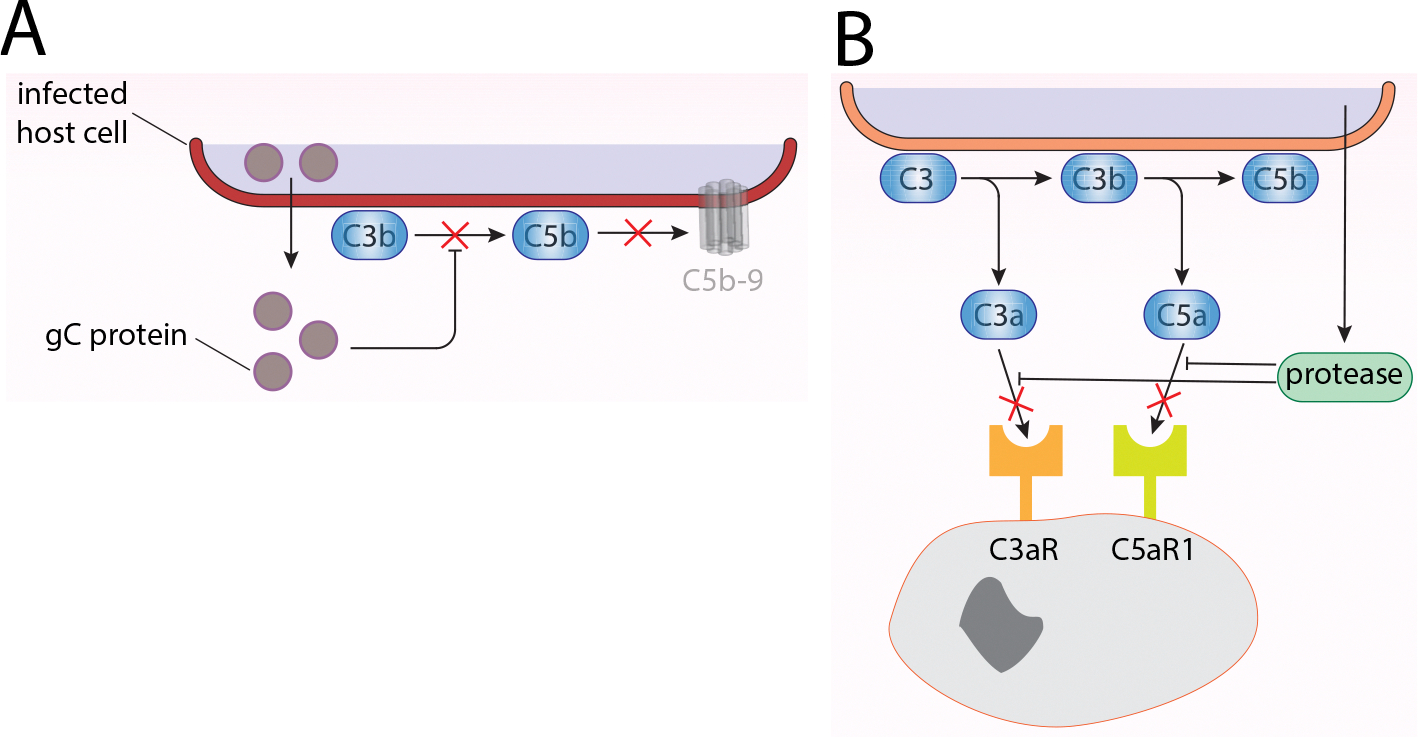
Avoidance of activation and function–inhibition. highly specific protein-protein interactions are the key feature of the complement cascade, and their abrogation is a common feature of pathogen evasion strategies. (A) A prominent example for this evasion strategy is the mature Herpes simplex virus (HSV) glycoprotein C (gC) that inhibits the activation of the complement cascade by binding C3b and by blocking binding of properdin and C5 to C3b, thereby interfering with the generation of the membrane attack complex (MAC) and the lysis of infected host cells. (B) The pathogen-derived complement-active proteases from *Entamoeba histolytica* and streptococci bacteria can degrade the anaphylatoxins C3a and C5a consequently blocking the activation of anaphylatoxin receptors C5aR and C3aR and their downstream assigned effector functions.

A viral example of the suppression of complement activation through C1q binding is the coat protein of human Astrovirus type 1 (HAstV-1) through the replacement of the C1rC1s tetramer (Bonaparte et al., 2008; Hair et al., 2010). Moreover, the HAstV-1 coat protein can bind MBL and therefore stops LP activation. Since the C3 convertase plays a central role in the cascade, it has become an obvious target for pathogenic invaders. Properdin (FP), is a positive AP regulator, is blocked from binding to bacterial surface by LPS (Ermert et al., 2019). The glycoprotein C (gC) of Herpes simplex virus 1 (HSV-1) impedes complement from two sites: one gC domain blocks FP and C5 binding to C3b, and another disables C3 and its fragments (Fig. 3A) (Fries et al., 1950; Hung et al., 1994; Kostavasili et al., 1950).

Inhibition of the LP is another target for human pathogens. For example, *T. cruzi* utilizes N- and O-glycosylated molecules on its surface to bind L- and H-ficolins, and MBL and thereby impedes MASP-1 and 2-induced C2 and C4 cleavage (Kahn et al., 1996; Cestari and Ramirez, 2010; Ribeiro et al., 2015). Additionally, the *T. cruzi* CRP binds the collagenous part of L-ficolin and inhibits the LP initiation (Sosoniuk et al., 2014).

#### 5.3.2. Interference with anaphylatoxin generation and MAC assembly

Interference with MAC formation represents the final opportunity for pathogens to evade complement-mediated lysis, and numerous microorganisms have evolved mechanisms to exploit this stage of the cascade. CspA is a surface-exposed protein of *B. burgdorferi* that inhibits the terminal complement pathway by directly binding to the complement components C7 and C9, thereby blocking MAC formation on the bacterial surface and in solution (Hallström et al., 2013). The *leptospiral* surface protein LcpA binds to the human serum component vitronectin and thereby suppresses polymerization of C9 (Fraga et al., 2016). The *Staphylococcus* super antigen-like 7 (SSL-7) protein prevents MAC formation at an earlier step: it binds C5 and blocks its interaction with the C5 convertase leading to a lack of C5b (Bestebroer et al., 2010).

Inactivating mechanisms of the anaphylatoxins are also present in bacteria, such as *Streptococcus* through ScpA (Lynskey et al., 2017). The protein chemotaxis inhibitory protein of *S. aureus* (CHIPS) inhibits chemotaxis of neutrophils and monocytes towards C5a by binding directly to C5aR1, thus preventing the chemotactic activity of C5a (Postma et al., 2004; de Haas et al., 2004).

MAC formation on virus-infected cells can lower the viral load. For instance, Zika virus inhibits MAC formation and complement-mediated lysis by binding terminal complement components to its viral E protein (Malekshahi et al., 2020).

The secreted protein Pra1 from *C. albicans* can bind C3a (as well as other C3 fragments) and prevent effector mechanisms (Luo et al., 2018).

The extracellular cysteine proteinase of the protozoa *Entamoeba histolytica* degrades the anaphylatoxins C3a and C5a, thus preventing the immunomodulatory effects of the anaphylatoxins (Fig. 3B) (Reed et al., 1950).

### 5.4. Employment – hijacking and mimicry of host complement components

#### 5.4.1. Employment of complement regulators for microbial protection

For the hosts, complement regulators are crucial for preventing damage of host tissue, hijacking of complement regulator functions are widely used as a mechanism of evasion (Table 4). Moreover, as many regulators share structural features, pathogens can synthesize a small arsenal to recruit various regulators, thus providing a low-cost solution to evading host’ complement (Lambris et al., 2008). One particularly common and shared mechanism is the employment of FH, FH like protein-1 and FH-related proteins to inhibit complement activation early in the cascade (Moore et al., 2021).

An alternative strategy to utilizing the host’ complement regulator arsenal is to encode proteins that mimic the function of host’ proteins. Mimicry of host molecules can be used in a protective way, an aggressive way, or both (Tables 5–7). The encoding of proteins homologous to components of regulators is a well-recognized viral strategy to subvert complement attack (Agrawal et al., 2020; Bernet et al., 2003). The recognition of viral mimicry dates to seminal work showing that the vaccinia virus (a member of the poxvirus family) encodes a secretory molecule resembling complement regulators (Kotwal and Moss, 1988).

**Fig. 4.**
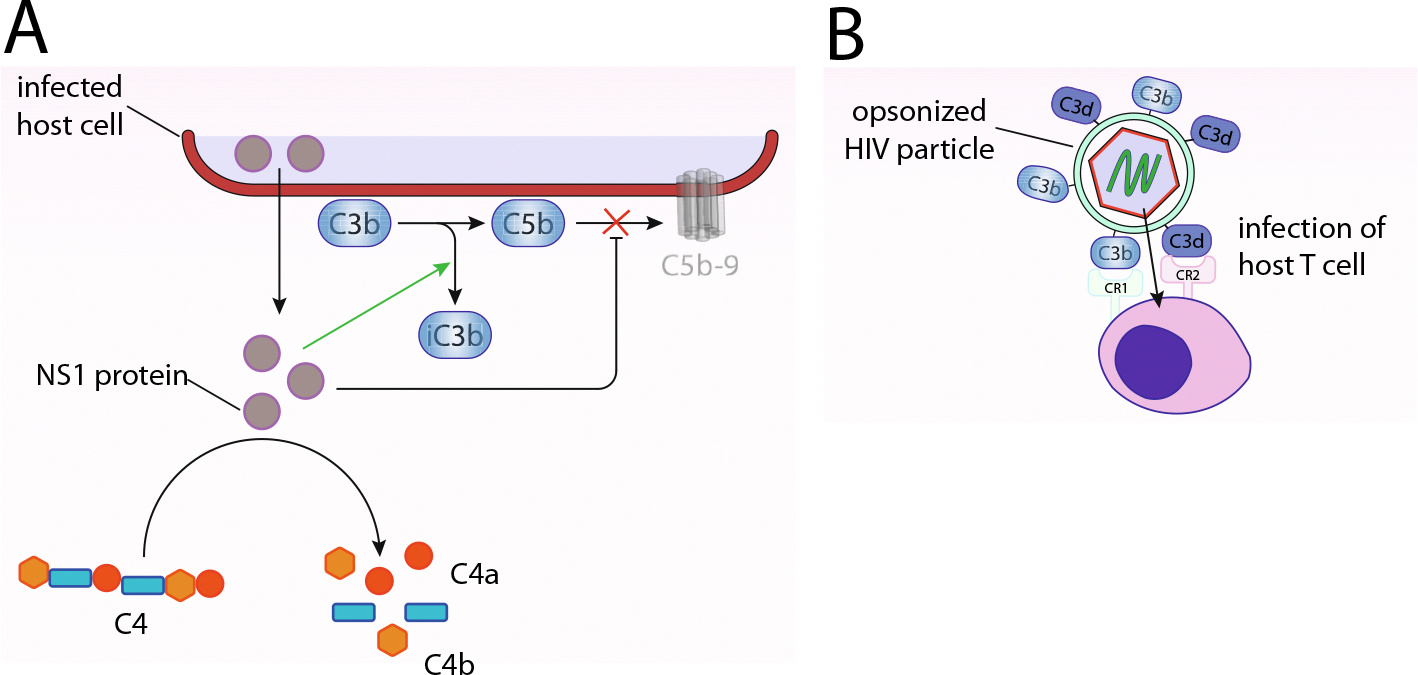
Employment–hijacking and mimicry of host complement components. (A) Hijacking of host regulatory proteins – Zika, Dengue, and West Nile viruses encode the NS1 protein which cleaves C4, and then binds and recruits factor H that result in C5b-9 (MAC) formation inhibition. (B) Expression of homologous protein of host regulatory proteins – opsonization of HIV-1 by complement leads to more effective infection of host T cells in a CR1- and CR2-dependent manner. CR1 facilitates the viral attachment to CR2 as CR1 acts as a cofactor for cleavage of C3b into C3d that is recognized by CR2.

Members of the flavivirus genus, including the Zika, Dengue and West Nile viruses, encode the (both secreted and membrane-bound) glycoprotein nonstructural protein 1 (NS1) which has many complement-modulatory effects (Conde et al., 2017; Mellors et al., 2020). These include the degradation of C4 (Avirutnan et al., 2010), binding and recruiting FH (Chung et al., 2006), inhibiting MAC formation by interacting with vitronectin (Conde et al., 2016), and the recruitment of C4BP (Fig. 4A) (Avirutnan et al., 1950). Nipah virus, which is resistant to *in-vitro* complement-mediated neutralization, possesses “FI-like” activity, which together with FH or CR1, can cleave C3b *in vitro* preventing C3 deposition on Nipah virus particles (Johnson et al., 2015). A similar FI-like activity and resistance to complement-mediated neutralization and C3 deposition was later observed in Chikungunya virus (Nag et al., 2020).

#### 5.4.2. Use of complement proteins for entry

Although the interaction of active complement components with their corresponding receptors on immune cells enables immune cells to hunt down pathogenic invaders, several pathogens have found a way to make use of complement receptors for their own purpose.

The binding of the host’s surface bound regulatory proteins, such as CD59, is a known mechanism to avoid MAC-mediated lysis in gram-negative bacteria (e.g., *Escherichia coli and Helicobacter pylori* (Rautemaa et al., 1998; Rautemaa et al., 2001)). Alternatively, instead of utilizing the host’s CD59, some strains of *B. burgdorferi* express a CD59-like protein protecting it from MAC activity. While both complement-sensitive and -resistant strains show similar levels of C3 deposition, resistant strains exhibit markedly reduced deposition of terminal complement components, especially C9 and poly-C9. This resistance is linked to a surface-expressed CD59-like molecule, identified as an ~80 kDa protein in resistant strains. Functionally analogous to human CD59, this molecule interferes with MAC assembly, primarily by binding C9 and partially C8 (Pausa et al., 2003).

The uptake of *Francisella tularensis*, a zoonotic intracellular bacterium and cause of potentially life-threatening tularemia, is mediated by CR3 and CR4 (Ben Nasr et al., 2006).

One of the most formidable examples of finding the right balance of complement activation and inhibition might be HIV-1. Opsonization of HIV-1 by complement results in more effective infection of CD4^+^ T cells with virus in a CR1- and CR2-dependent manner (Fig. 4B) (Thieblemont et al., 2008). In addition to linking opsonized HIV to CD4^+^ T cells, CR1 facilitates the viral attachment to CR2 as CR1 acts as a cofactor for cleavage of C3b into C3d which is recognized by CR2 (Delibrias et al., 1993).

The Epstein-Barr virus (EBV) expresses the glycoprotein gp350/220 that interacts with CR1 and majorly CR2 as cell entry receptor on B cells and immature T cells leading to viral infection and manifestation of infectious mononucleosis (kissing disease) (Fingeroth et al., 1984; Ogembo et al., 2013).

*C. albicans* also contains one or more CR3-like molecules which facilitate adhesion to host cells (Gale et al., 1996; Hostetter, 2008) of which some are binding to FH (REF). In this case, it is not the human complement receptor on the hosts cell, but the fungal replicate of that molecule interacting with host complement components.

CR1 serves as major receptor on macrophages for the intracellular protozoan parasite *Leishmania major* (Da Silva et al., 1950; Rosenthal et al., 1996). Moreover, CR3 and CR4 have been reported in contributing to the phagocytosis and survival of the tropical parasites *P. falciparum* in erythrocytes (Zhou et al., 2015) and *Leishmania spp*. in macrophages (Wilson and Pearson, 1988; Mosser et al., 1992).

#### 5.4.3. Employment of complement for both protection and attack

FH binding to the surface of certain pathogens does not only protect from destruction, e.g. via impairment of phagocytosis and killing by neutrophils, as shown for *C. albicans* (Kenno et al., 2018), but may also enhance adherence and promotes invasion (e.g. as seen in S*. pneumoniae* (Hammerschmidt et al., 2007), *Mycoplasma hyopneumoniae* (Yu et al., 2020), and *A. fumigatus* (Dasari et al., 2019)).

## 6. The new era of complement biomarkers

### 6.1.1. Specific assessment of complement proteins and their activation as a prerequisite

Having detailed the many effector mechanisms through which complement limit infections, and having deciphered the multiple evasion mechanisms that pathogens possess to evade complement, several ideas will be presented below on how to use complement related molecules as biomarkers or therapies.

As a prerequisite, it is important to determine which part of the complement system is activated. This started about three decades ago with the concept of testing the increase of complement activation molecules as biomarkers (either split products of whole complement proteins, such as C5a, or complexes thereof, such as C5b-9) rather than the studying decrease in the respective native molecules (such as C4 and C3). This was made possible by the use of neoepitope-specific monoclonal antibodies that react solely with the activated version of the molecule, in contrast to monoclonal antibodies that would bind to the non-activated state (Oppermann and Würzner, 2010). Neoantigen antibodies such as aE11b (Mollnes et al., 1985), WU 13–15 (Würzner et al., 1991) and bh6 (Garred et al., 1988) have revolutionized the detection of activated complement components. The use of these antibodies has been extremely useful in detecting residual functionally active complement components in subtotal deficiencies (Würzner et al., 1995; Würzner et al., 1996).

Good biomarkers for activation of complement following infectious disease should be based on specific monoclonal antibodies. The best profile for them would be easy to use, affordable, and prognostic, and should be capable of monitoring the disease, detecting active flares and possibly to tailor treatment. While all this appears to be achievable, a biomarker for a specific pathogen or disease likely remains music of the future. Thus, a lot of complement activation markers points towards complement activation but are usually not specific for an individual disease (Ekdahl et al., 2018; Cohen et al., 2012; Wurzner, 2023).

There are however some examples which could fulfill the requirements of being useful tools in diagnostic purposes. For example, the measurement of C1q in tuberculosis. C1q is only elevated in active tuberculosis, making C1q a useful tool in determining active vs dormant tuberculosis infection (Lubbers et al., 2018). Another example is the concentration of pentraxin, which is associated with severity of SARS-CoV 2 infection, and has shown prognostic value in predicting survival (Assandri et al., 2022). However, neither C1q nor pentraxin are specific for these infections, yet they could still represent useful tools in the clinic.

Promisingly, there are recent developments of ELISAs which can specifically monitor the CP or LP by measuring either the C1s-C1 inhibitor complex or the MASP2-C1 inhibitor complex (Hurler et al., 2022). C1s-C1 inhibitor complexes have been shown to be relevant in multiple clinical scenarios, such as HIV-positive patients (Füst et al., 1991), systemic lupus erythematosus (Auda et al., 1990), rheumatoid arthritis and glomerulonephritis (Waldo and West, 1987), indicating great clinical promise for these assays.

### 6.1.2. Complement targeting therapies in treatment of infections

The use of complement-targeting drugs has been proposed for some time now. In 2009, shiga toxin was discovered to activate complement and bind to FH (Orth et al., 2009). This leads to complement activation being seen in haemolytic uraemic syndrome (HUS) patients (Orth and Würzner, 2010). It was therefore proposed that complement inhibitory therapy may limit kidney destruction, and three index cases indeed recovered miraculously well with anti-C5 therapy (Lapeyraque et al., 2011). This lead to a humanized anti-C5 antibody (Eculizumab) being used in a big HUS outbreak in Northern Germany in 2011, caused by contaminated fenugreek sprouts (Orth-Höller et al., 2011). Nowadays this treatment is still recommended for severe forms of the disease with neurological involvement (Wildes et al., 2024).

Also recently, SARS-CoV 2 has been proposed to activate complement, thus, Eculizumab was tried in clinical trials (Peffault de Latour et al., 2020; Annane et al., 2020; Ruggenenti et al., 2021; Burwick et al., 2022) however, not resulting in a clear recommendation (Zelek and Harrison, 2023). It must be kept in mind that Eculizumab treatment itself can lead to adverse outcomes, such as infection, with meningococcal disease being particularly common (Dhanoa et al., 2022). This is not surprising as Eculizumab targets the terminal pathway and terminal pathway deficiencies normally cause this type of infection (Figueroa and Densen, 1991). As activation of SARS-CoV 2 is thought to occur mainly via the LP (Eriksson et al., 2020; Götz et al., 2022) a MASP-2 inhibitor seemed to work much better (Flude et al., 2021). At present no drug interfering with the complement system can be recommended for the treatment of SARS-CoV 2.

However, maybe the most striking example of use of microbial evasion mechanisms in human medicine is not in regard to the treatment of an infection, but rather with the prevention of infection. *Meningococcus* FH binding protein is such an important evasion molecule that this has been successfully been translated for the use in a vaccine (Principato et al., 2020).

## 7. Conclusion

The host’s complement system is among the first and most potent barriers that pathogens encounter when infecting a human host. As such, evolutionary pressures have led to a diverse and potent set of strategies to counteract the complement effector functions. Future considerations that target complement as a possible therapeutic must consider these evasion techniques as a barrier to success. Furthermore, one must consider not only the complement system in isolation but the dynamic battle between host and pathogen, where location, timing and several other factors are at interplay.

Moreover, too much or too little complement activation in infectious diseases is potentially harmful to the host, particularly in infectious diseases. Thus, novel strategies in fighting infectious diseases should always consider that the microbe itself can not only evade complement to prevent its own destruction, but may also use it for invasion into host cells.

The repetitive manner through which viruses, bacteria, fungi and protists all target the complement system highlights the importance of complement in preventing infections. Through this review we aimed to provide a platform through which future diagnostic and therapeutic tools can be developed to help patients around the world.

## Figures and Tables

**Table 1:** Structural barriers.

Microbial class	Species	Mechanism	Complement target	Effect	Literature

Virus	SARS-CoV-1/-2	Epitope masking	Likely CP activation	“Down” conformation masks epitope from antibody recognition	(Yuan et al., 2020)
	Herpesviridae and Coronaviridae	Steric hindrance by binding non-specific antibodies	Likely CP activation	Steric hindrance by binding non-specific antibodies	(Dowler and Veltri, 1984; Frank and Friedman, 1989)
	HIV-1	Glycosylation of surface	Likely CP activation	Glycosylation prevents recognition by host’s antibodies, preventing CP activation	(Rathore et al., 2017)
Fungi	*A. fumigatus*	Prevention of complement deposition	Complement fragment deposition	Melanin might mask complement-activating moieties on the surface of fungus	(Speth and Rambach, 2012; Tsai et al., 1997; Tsai et al., 1998)
Bacteria	*Neisseria meningitidis*	Prevention of C3b deposition and activation of CP	Inhibits CP and amplification loop	Capsule prevents activation of CP and deposition of C3b, thus preventing both CP and amplification loop.	(Agarwal et al., 2014; Roberts, 1996)
	*Neisseria gonorrhea*	Sialylation of LOS inhibits all activation pathways	Inhibits all activation pathways	Sialylation of LOS interferes with IgG binding, MBL recognition and represses AP activation through enhanced FH binding and therefore inhibits all three complement pathways	(Elkins et al., 1992; Gulati et al., 2002)
	*Streptococcus pyogenes*	Binding of human proteins to M-protein to avoid complement activation	Inhibits all activation pathways	M-protein interacts with many human proteins such as FH, IgG Fc domain and albumin to prevent complement deposition and phagocytosis	(Whitton and Feuer, 2004)
Protist	*Leishmania spp.*	Inhibition of MAC mediated killing by surface	MAC mediated killing	Disruption of glycocalyx inhibits susceptibility to MAC-mediated killing	(Puentes et al., 1950; Späth et al., 2003)

**Table 2:** Shedding or destruction.

Microbial class	Species	Mechanism	Complement target	Effect	Literature

Virus	Alphaherpesvirus Pseudorabies virus,	Clearance of antibody-antigen complex	CP activation	Clearance of antibody-antigen complex prevents recognition by CP	(Favoreel et al., 1999)
	Flavivirus	Promoting cleavage of C4	C4	NS1 promotes cleavage of C4 into C4b, and also promotes the recruitment of C4BP, inactivating C4.	(Avirutnan et al., 1950)
	RSV Rhinovirus, Polio 2 Virus	C3 degrading proteases	C3	C3 degrading proteases inhibits intracellular opsonization by complement.	(Tam et al., 2014)
Bacteria	*K. pneumoniae*	Removal of sugar moieties	LP recognition	Removal of sugar moieties on capsule prevents recognition by LP	(Sahly et al., 2009)
	*Pseudomonas spp.*	Pathogen proteases cleaving complement	C1q	Cleavage of C1q targets the CP	(Rooijakkers and Van Strijp, 2007)
	*Salmonella spp.*	Shedding complement from the pathogen’s surface	Assembling MAC	Complement activation impaired	(Joiner et al., 1982)
	*Streptococcus pyogenes*	Degradation of C3 by protease Streptococcal pyrogenic exotoxin B	C3	Impairs opsonization and all complement pathways	(Kuo et al., 2008)
	*Streptococcus pyogenes*	Release of complement-degradating proteins from pathogen surface	C5a	Releases a C5a peptidase, inhibiting chemotactic potential of C5a.	(Berge and Björck, 1995)
	*Neisseria meningitidis*	NalP cleaves C3	C3 and downstream opsonization	Impairs opsonization and the anaphylatoxin effects of C3a.	(Del Tordello et al., 2014)
	*Staphylococcus aureus*	Serine protease-like protein B (SplB) cleaves C3-C9, and FB and activation fragments of C3 and C4	C3-C9, FB and activation fragments of C3 and C4.	Blocks all complement pathways	(Dasari et al., 2022)
Fungus	*Aspergillus fumigatus*	Alp1 degrades C1q, C3, C4, C5, MBL, FD	C1q, C3, C4, C5, MBL, FD	Reduced opsonization and phagoyctosis.	(Rambach et al., 2010; Behnsen et al., 2010)
Protist	*Trypanosoma spp.*	Clearance of antibody-antigen complex	CP activation	Clearance of antibody-antigen complex prevents recognition by CP	(Balber et al., 1979; Frevert and Reinwald, 1990)
		Cleavage of C3b	Removal of C3b from surface	Binding of C3b to complement regulator y protein renders it susceptible to cleavage	(Norris, 1996b)

**Table 3:** Recruitment for protection.

Microbial class	Species	Mechanism	Complement target	Effect	Literature

Virus	Sindbis Virus	Sialic acid on pathogen surface	FH recruitment	Quantity of sialic acid and subsequent recruitment of FH influence AP activity	(Hirsch et al., 1983; Hirsch et al., 1950)
	HIV-1	CD46, CD55 and CD59 incorporated into HIV-1 particles and function to protect virions from complement-mediated destruction	CD46, CD55, CD59	Protects from complement mediated destruction by complement-regulating mechanisms	(Montefiori et al., 1994; Saifuddin et al., 1995)
	Human Cytomegalovirus	Upregulation of CD46 and CD55 on the infected cells	CD46, CD55	Inhibition of all pathways	(Spiller et al., 1996)
	Vaccinia virus	Incorporation of CD46, CD55, and CD59 into the outer envelope	CD46, CD55, CD59	Inhibition of all pathways	(Vanderplasschen et al., 1998)
Bacteria	*Neisseria gonorrhoeae*	Glycosylation of pathogen surface	FH	Glycosylation enhances binding of FH to pathogen surface, resulting on inhibition of complement by FH	(Ram et al., 1998; Vimr et al., 2004)
	*Pseudomonas aeruginosa, Mycoplasma hyopneumoniae*	Binding of host’ FH and/or plasminogen	FH	Binding of FH and plasminogen leads C3b degradation and cleavage of plasminogen substrates	(Kunert et al., 2007; Yu et al., 2020)
	*Borrelia burgdorferi*	Binding of FH, FHL-1, plasminogen	FH, FHL-1, plasminogen, C7, C9	Enhances FH-mediated C3 inactivation, inhibits MAC, and degrades plasminogen substrates	(Hart et al., 2018; Siegel et al., 2008; Hartmann et al., 2006)
		Binding of FH, FH-L1 with complement regulator acquiring surface proteins	FH, FHL-1	Inhibition of C3 activation & C3 convertase formation, termination of the assembly and finally the integration of the MAC into the bacterial membrane	(Locke, 2019)
	*Acinetobacter baumannii*	Recruitment of FI leads to formation of complex and degradation of C3b and C4b	C3, C3b, C4b, C5, FB, FD & esp. FI	Inhibition of all three complement pathways.	(Ries et al., 2022)
	*Leptospira sp.*	Once bound to the leptospiral surface, FH and C4BP retain cofactor activity of FI in the cleavage of C3b and C4b	FH, FH-like-1, C4BP, Vitronectin	Binding of vitronectin suppresses polymerization of C9	(Fraga et al., 2016)
Fungus	*Candida albicans, Aspergillus fumigatus*	Recruitment of FH and other complement regulators to surface	FH, FHL-1, C4BP, plasminogen	Avoids activation of complement	(Kenno et al., 2018; Dasari et al., 2019; Meri et al., 2002; Luo et al., 2011)
Protist	*Trypanosoma cruzi*	Transfer of host cell sialic acids	Promotes negatively charged surface inhibiting AP.	Avoids activation of complement-mediated lysis and enhances intracellular invasion	(Tomlinson et al., 1950; Weatherly et al., 2016; Dc-Rubin and Schenkman, 2012)
	*Plasmodium spp.*	Recruitment of C1-INH to the surface PfMSP3.1 protein	C1-INH	Inhibition of activating proteases of the complement cascade C1s, MASP1, and MASP2	(Kennedy et al., 1950)
	*Plasmodium falciparum*	Glideosome-associated protein 50 (GAP50) binds to FH	FH,	Binds FH and uses surface-bound FH to inactivate C3b	(Schmidt et al., 2015; Simon et al., 2013)
		Inhibition of terminal complement pathway by CD59 recruitment	PfPIG-M	Prevention of MAC formation on pathogen-infected red blood cells	(Kiyuka et al., 2020; Rashidi et al., 2022; Wiesner et al., 1997; Kim and Hong, 2007)
		Inhibition of C3-convertases by C4BP recruitment	Circumsporozoite protein (CSP)		(Khattab et al., 2022)
	*Trichomonas vaginalis*	Inhibition of terminal complement pathway by CD59 recruitment	unknown	Prevention of MAC formation and pathogen lysis	(Ibáñez-Escribano et al., 2015)
	*Leishmania species*		C4bBP receptor	Acceleration of C3-convertases decay	(Pereira-Filho et al., 2023)
	*Entamoeba histolytica*	Recruitment of CD46, CD55, and CD59 by trogocytosis		Inhibition of all three pathways	(Miller et al., 2022)
	*Toxoplasma gondii*	Recruitment of C4BP, FH to the surface to inactivate surface-bound C3 and limit formation of the MAC	FH, C3b, C4BP	Blockade of AP amplifies complement activation & killing of T. gondii	(Rashidi et al., 2022; Sikorski et al., 2020; Sikorski et al., 2021)

**Table 4:** Recruitment for aggression.

Microbial class	Species	Mechanism	Complement target	Effect	Literature

Bacteria	*Streptococcus pneumoniae*	FH attaches to the surface of pathogen	FH	Enhances adherence to host cells	(Hammerschmidt et al., 2007)
Fungus	*A. fumigatus*	*Aspergillus* secreted galacosaminogalactant leads to complement deposition on platelets.	Complement activation on surface of platelets	Complement deposition on surface of platelets leads to reduced platelet viability, perhaps due to increased MAC formation on the surface of platelets.	(Deshmukh et al., 2020)
Protist	*Leishmania spp.*	C3b conversion to iC3b prevents C3 convertase formation on the parasite surface, surface deposited iC3b is recognized by CR3/MAC-1, resulting in parasite phagocytosis by macrophages	C3b/iC3b	Decreased lytic activity but increased opsonization of pathogen.	(Cestari et al., 2013; Brittingham et al., 1950; Russell, 1987)

**Table 5:** Mimicry for protection.

Microbial class	Species	Mechanism	Complement target	Effect	Literature

Virus	VACV	Homologous protein of host regulatory proteins	C3b; C4b	Binds C3b and C4b and accelerates decay of CP/LP as well as AP C3 convertases (“decay-accelerating activity”), supports inactivation of C3b and C4b with the help of FI (“cofactor activity”), avoids antibody-mediated & complement dependent virus neutralization	(Kotwal and Moss, 1988; McKenzie et al., 1992; Kotwal et al., 1990)
	Zika, Dengue, West Nile Virus		NS1	Degradation of C4 binding and recruitment of FH, inhibition of MAC formation by interaction with vitronectin, recruitment of C4BP	(Conde et al., 2017; Mellors et al., 2020; Avirutnan et al., 2010; Chung et al., 2006; Conde et al., 2016; Avirutnan et al., 1950)
Bacteria	*E. coli, H. pylori,*		CD59	Mechanism of evasion of MAC-mediated lysis	(Rautemaa et al., 1998; Rautemaa et al., 2001)
	*Borrelia burgdorferi*		CD59-like protein	Mechanism of evasion of MAC-mediated lysis	(Pausa et al., 2003)
Fungi	*Aspergillus*		CD59	Mechanism of evasion of MAC-mediated lysis	(Vogl et al., 2008)
Protist	*Schistosoma mansoni, Fasciola hepatica*		C8, C9	Binds to human C8 and C9, and inhibits the assembly of MAC	(Farias et al., 2013; Shi et al., 2014; Castro-Borges et al., 2011; Wilson and Coulson, 2009; Parizade et al., 1994)
	*Trypanosoma cruzi*	Expression of T-DAF	C3b; C4b	Accelerates the dissociation or assembly efficiency of C3 convertases	(Kipnis et al., 1988; Tambourgi et al., 1993; Cardoso et al., 2015; Joiner et al., 1988; Rimoldi et al., 1988; Tambourgi et al., 1995)
	*Plasmodium falciparum*	Inhibition of terminal complement pathway by CD59 recruitment	PfPIG-M	Prevention of MAC formation on pathogen-infected red blood cells	(Kiyuka et al., 2020; Rashidi et al., 2022; Wiesner et al., 1997; Kim and Hong, 2007)
		Inhibition of C3-convertases by C4BP recruitment	Circumsporozoite protein (CSP)		(Khattab et al., 2022)
		Expression of PfGPI protein	C1-INH	Inhibition of the CP	(Rashidi et al., 2022; Touray et al., 1994; Mejia et al., 2016)
	*Trichomonas vaginalis*	Inhibition of terminal complement pathway by CD59 recruitment	unknown	Prevention of MAC formation and pathogen lysis	(Ibáñez-Escribano et al., 2015)
	*Leishmania species*		C4bBP receptor	Acceleration of C3-convertases decay	(Pereira-Filho et al., 2023)

**Table 6:** Mimicry for aggression.

Microbial class	Species	Mechanism	Complement target	Effect	Literature

Virus	Vaccinia virus	Vaccinia virus complement protein (VCP) binds C3b and C4b, accelerates decay of classical/lectin and alternative pathway C3 convertases, and acts as a cofactor for FI-mediated inactivation of C3b and C4b, thereby preventing antibody- and complement-dependent virus neutralization. Orthologs with similar complement inhibitory functions are found in variola, monkeypox, cowpox, and ectromelia viruses	C3b, C4b	Acts in similar way to C4bBP and is structurally similar to CR1, resulting in decreased complement activation.	(McKenzie et al., 1992; Kotwal et al., 1990; Jha and Kotwal, 2003; Rosengard et al., 2002; Sfyroera et al., 2005; Yadav et al., 2008; Yadav et al., 2012; Liszewski et al., 2006; Chen et al., 2005; Likos et al., 2005; Hudson et al., 2012; Miller et al., 1997; Moulton et al., 2010; Sahu et al., 1998; Isaacs et al., 1992; Smith et al., 2000; Girgis et al., 2008; DeHaven et al., 2010)
Fungus	*C. albicans*	Expression of homologous host complement proteins	iC3b	CR3-like molecule facilitates adhesion to host cells and is a virulence factor; (mimicry of that molecule on the pathogen site, interacting with other host ligands)	(Gale et al., 1996; Hostetter, 2008)

**Table 7:** Other mechanisms.

Microbial class	Species	Mechanism	Complement target	Effect	Literature

Virus	HSV-1	gE and gI, which together form a heterodimeric complex and function as IgG Fc receptor (FcγR) that then participates in immune evasion by promoting ‘antibody bipolar bridging’	CP	Prevents CP activation	(Frank and Friedman, 1989; Johnson and Feenstra, 1987; Johnson et al., 1988; Dubin et al., 1991; Lubinski et al., 2011; Sprague et al., 2006; Awasthi et al., 2014)
	HCV	Transcriptional repression of complement molecules	C2, C3, C4, C9	Inhibition of the CP/LP C3 convertase formation	(Kim et al., 2014; Kim et al., 2013; Mazumdar et al., 2012; Banerjee et al., 2011)
	Human astroviruses	Replacement of the C1rC1s tetramer or binding to MBL impedes CP/LP activation	C1/MBL	Reduced CP/LP activation.	(Bonaparte et al., 2008; Hair et al., 2010)
	Zika Virus	Binding of the E Protein to components of the terminal pathway complement	C5b, C6, C7, C8, C9	Protein interferes with the polymerization of C9, reduces MAC formation by binding terminal complement proteins	(Malekshahi et al., 2020)
	HIV-1	CR1 facilitates the viral attachment to CR2 as CR1 acts as a cofactor for cleavage of C3b into C3d that is recognized by CR2	CR1, CR2	Enhanced invasion of T cells by virus	(Delibrias et al., 1993)
	EBV	gp350/220 interacts with CR1 and majorly CR2 that leads to viral infection	CR1, CR2	Enhanced invasion	(Fingeroth et al., 1984; Ogembo et al., 2013)
Bacteria	*K. pneumoniae*	Removal of sugar moieties from capsule	LP	Removal of sugar moieties on capsule prevents recognition by LP	(Sahly et al., 2009)
	*Borrelia sp.*	Prevents C9 polymerization, TCC assembly, and prevents integration of the functional pore-forming complex	C5, C7, C9, MAC	Reduced MAC formation	(Hallström et al., 2013; Locke, 2019; Marcinkiewicz et al., 2017; Hammerschmidt et al., 2016)
	*Haemophilus influenzae, Staphylococcus aureus, Streptococcus pneumoniae, Borrelia burgdorferi, Leptospira interrogans*	Acquisition of host’ plasminogen and/or vitronectin	Plasminogen substrates	e.g Cleavage of C3b	(Barthel et al., 2012; Brissette et al., 2009; Bergmann et al., 2004; Koch et al., 2012; Vieira et al., 2010; Vieira et al., 2009)
	*Staphylococcus aureus,*	Chemotaxis inhibitory protein of Staphylococcus aureu (CHIPS) binds to C5a and its receptor	C5aR1	Prevents chemotactic activity of C5a	(Postma et al., 2004; de Haas et al., 2004)
	*Staphylococcus aureus*	Abrogates interaction of C5 with C5 convertase	C5	Blocks MAC formation	(Bestebroer et al., 2010)
	*Francisella tularensis*	Complement receptors facilitate intraceullar invasion of pathogens and intracellular survival	CR3, CR4	Enhances intracellular invasion and survival	(Ben Nasr et al., 2006)
	*Streptococcus pyogenes, Streptococcus pneumoniae*	Complement activation by soluble proteinslead to complement activation far away from surface	Complement factors depletion	e.g. reduced opsonization of pathogen	(Berge et al., 1997; Mitchell and Andrew, 1997)
Fungus	*Candida albicans*	Acquisition of host’ plasminogen, FH and FHL-1	C3b and plasminogen substrates	Enhances FH-mediated inactivation as well as degradation of plasminogen substrates	(Luo et al., 2009)
		Pra1 targets and blocks C3 and C3 activation fragments	C3, C3a	Pra1 cleaved C3 at a unique site and further inhibited effector function of the activation fragments Abrogates C3a effector mechanisms through binding of C3a	(Luo et al., 2018)
Protist	*Plasmodium falciparum*	Binds human nonimmune IgM and occupies C1q-binding sites on IgM and hence prevent complement fixation	C1q	Prevents complement fixation.	(Simon et al., 2013; Kiyuka et al., 2020; Mejia et al., 2016; Rasti et al., 2006; Akhouri et al., 2016; Larsen et al., 2019; Barfod et al., 2011)
	*Haemonchus contortus*	The parasite enzyme GAPDH binds to C3 and C1q and inhibits their activity	C3, C1q	Prevents MAC formation and release of anaphylatoxins	(Sahoo et al., 2013; Vedamurthy et al., 2015)
	*Trypanosoma brucei gambiense*	ISG65 as a receptor for human C3 and its activation fragments – takes over a role in selective inhibition of the AP C5 convertase	C3, C3b	Abrogation of the terminal pathway	(Sülzen et al., 2023)
	*Trypanosoma sp.*	Inhibits formation of cell-bound and fluid-phase AP C3, CP and possibly MBL-mediated LP activation. Also inhibits the cleavage of C2 by C1s or MASP-2 and consequently prevents C3 convertase formation by competing with C4.	H-/L-ficolins, MBL, C1q, C4	Inhibition of overall complement activation.	(Norris, 1996b; Kahn et al., 1996; Cestari and Ramirez, 2010; Ribeiro et al., 2015; Sosoniuk et al., 2014; Cestari et al., 2013; Cardoso et al., 2015; Ferreira et al., 2004a; Ferreira et al., 2004b; Ramírez-Toloza et al., 2020; Cestari et al., 2008; dos Cestari et al., 2009; Inal et al., 2005; Inal and Schifferli, 2002; Beucher and Norris, 2008; Oladiran and Belosevic, 2010; Aguilar et al., 2005; González et al., 2015; Ramírez et al., 2011; Ramírez et al., 2012; Ramos et al., 1991; Aguillón et al., 1997; Rimoldi et al., 1989; Engstler et al., 2007; Rudenko, 2011; Fischer et al., 1988; Ouaissi et al., 1986; Ouaissi et al., 1984; Velge et al., 1988; Ouaissi et al., 1988; Norris et al., 1989; Norris et al., 1950; Norris and Schrimpf, 1994; Norris et al., 1997; Norris, 1998; Henrique et al., 2016)
	*Plasmodium falciparum, Leishmania spp.*	Complement receptors facilitate binding an invasion of pathogen	C1, CR3, CR4	Enhances intracellular invasion.	(Da Silva et al., 1950; Rosenthal et al., 1996; Wilson and Pearson, 1988; Mosser et al., 1992)

## Data Availability

No data was used for the research described in the article.
